# Genome Sequence of a Beak and Feather Disease Virus from an Unusual Novel Host, Australian Boobook Owl (Ninox boobook)

**DOI:** 10.1128/mra.00172-22

**Published:** 2022-03-23

**Authors:** Subir Sarker, Ajani Athukorala, David N. Phalen

**Affiliations:** a Department of Microbiology, Anatomy, Physiology and Pharmacology, School of Agriculture, Biomedicine and Environment, La Trobe University, Melbourne, VIC, Australia; b Sydney School of Veterinary Science, Faculty of Science, The University of Sydney, Camperdown, NSW, Australia; c Schubot Exotic Bird Health, Texas A&M College of Veterinary Medicine and Biomedical Sciences, College Station, Texas, USA; Portland State University

## Abstract

The beak and feather disease virus (BFDV) is a pathogen of psittacine birds. BFDVs infecting nonpsittacine birds remain largely uncharacterized. We report the genome of a BFDV from a boobook owl (Ninox boobook), a nonpsittacine bird. The genome consisted of 1,993 bp containing two major bidirectionally transcribed open reading frames.

## ANNOUNCEMENT

Beak and feather disease virus (BFDV) is a member of the family *Circoviridae*. Like other circoviruses, BFDV possesses a circular single-stranded, approximately 2.0-kb DNA genome that is encapsidated into a nonenveloped, spherical icosahedral virion (1), and it contains two bidirectionally transcribed genes. BFDV infection was thought to be restricted to Psittaciformes (2–6), but evidence of infection in distantly related Australian avian species was demonstrated in the rainbow bee-eater (Merops ornatus) (7), powerful owl (Ninox strenua) (8), and finches (9). Many other nonpsittacine birds are also likely susceptible to sporadic spillover infection (10). Here, we report the characterization of a BFDV genome in a nonpsittacine bird, the boobook owl (Ninox boobook), a species of Strigiformes.

Kidney tissue was collected from a dead boobook owl (*Ninox boobook*) submitted to the Avian, Reptile, and Exotic Pet Hospital of the University of Sydney, Camden Campus (34°0′10.61″S, 150°37′27.84″E), between December 2018 and April 2019. Total genomic DNA was extracted using a PureLink genomic DNA minikit (Invitrogen, CA). The library was prepared using Illumina DNA prep (Illumina, San Diego, CA), starting with 250 ng of DNA (11). The quality and quantity of the prepared library were assessed by the Australian Genome Research Facility, Melbourne, Australia, and the library was sequenced using the Illumina NovaSeq sequencing platform, generating 150-bp paired-end reads.

Sequencing data were analyzed as per established pipeline (12–15) using Geneious (version 10.2.2; Biomatters, New Zealand) and CLC Genomics Workbench (version 9.5.4). Briefly, a total of 37,770,262 raw reads were preprocessed to remove the Illumina adapter, ambiguous base calls, and poor-quality reads (trim using quality score, limit 0.05; trim ambiguous nucleotides up to 15 using CLC Genomics Workbench), followed by mapping against barn owl (Tyto alba) (16) and Escherichia coli (GenBank accession no. U00096) to remove nonviral DNA. A total of 37,612,162 trimmed and unmapped reads were used as input data for *de novo* default assembly in CLC Genomics Workbench (version 9.5.4). This resulted in the generation of a 1,993-bp BFDV genome with an average coverage of 39.61×. Annotation and circularization of the assembled genome were performed using in Geneious (version 10.2.2). All software was used with default parameters except where stated.

The genome has 1,993 bp, with a G+C content of 53.8%. A BLASTn analysis (under GenBank database parameters, maximum target sequences: 100) (17) of the sequenced BFDV genome in this study showed an overall 78.75 to 98.54% pairwise identity with other BFDV genomes, showing highest sequence similarity to BFDV (98.54%) from a little corella (Cacatua sanguinea) from Australia (GenBank accession no. KY189060.1). In addition, when we compared a segment of capsid gene of BFDV sequenced (∼407 bp) in this study, we found a 82.3% pairwise identity with a previously sequenced partial capsid gene from a boobook owl (*Ninox boobook*) (GenBank accession no. KY410375.1) (10). The genome architecture of boobook BFDV sequence had characteristics of other *Circoviridae*, including two major open reading frames (ORFs): ORF1, encoding a replication-associated protein (293 amino acids), and ORF2, encoding the capsid protein (247 amino acids).

This study provides evidence of BFDV infection in an Australian boobook owl, as an unusual nonpsittacine host. Its apparent origin from a corella host suggests that the infection occurred from a contaminated nest hollow.

### Data availability.

The complete beak and feather disease virus genome sequence of *N. boobook* has been deposited in DDBJ/ENA/GenBank under accession no. OL762453. The version described in this paper is the first version, OL762453.1. The data that support the findings of this study are accessible via GenBank accession no. OL762453. The raw sequencing data from this study have been deposited in the NCBI Sequence Read Achieve (SRA) under accession no. SRR17163735 (BioProject accession no. PRJNA787018).
